# Thermal Conductivity Enhancement of Doped Magnesium Hydroxide for Medium-Temperature Heat Storage: A Molecular Dynamics Approach and Experimental Validation

**DOI:** 10.3390/ijms252011139

**Published:** 2024-10-17

**Authors:** Anti Kur, Jo Darkwa, Mark Worall, John Calautit, Rabah Boukhanouf

**Affiliations:** Buildings, Energy and Environment Research Group, Faculty of Engineering, University of Nottingham, University Park, Nottingham NG7 2RD, UK; anti.kur@nottingham.ac.uk (A.K.); lazmw@exmail.nottingham.ac.uk (M.W.); ezzjkc@exmail.nottingham.ac.uk (J.C.); lazrb@exmail.nottingham.ac.uk (R.B.)

**Keywords:** thermochemical heat storage, magnesium hydroxide, thermal conductivity enhancement, molecular dynamics simulations, medium-temperature heat storage, nanomaterials

## Abstract

Magnesium hydroxide, Mg(OH)_2_, is recognized as a promising material for medium-temperature heat storage, but its low thermal conductivity limits its full potential application. In this study, thermal enhancement of a developed magnesium hydroxide-potassium nitrate (Mg(OH)_2_-KNO_3_) material was carried out with aluminum oxide (Al_2_O_3_) nanomaterials. The theoretical results obtained through a molecular dynamics (MD) simulation approach showed an enhancement of about 12.9% in thermal conductivity with an optimal 15 wt% of Al_2_O_3_. There was also close agreement with the experimental results within an error of ≤10%, thus confirming the reliability of the theoretical approach and the potential of the developed Mg(OH)_2_-KNO_3_ as a medium heat storage material. Further investigation is, however, encouraged to establish the long-term recyclability of the material towards achieving a more efficient energy storage process.

## 1. Introduction

In the shift towards more sustainable energy systems, the significance of energy storage cannot be overstated. Therefore, thermal energy storage (TES) is recognized as a pivotal element in bridging the gap between heat demand and supply and serves as a means to harness and reuse otherwise wasted heat [1]. In this context, thermochemical energy storage (TCES) in inorganic hydroxide/oxide systems, with the advantage of higher energy density and minimal heat losses in storage, promises more compact energy storage in the short and long term.

Medium-grade heat (200 °C to 500 °C) accounts for a vast proportion of waste heat available in the commercial and industrial sectors. One potential TCES material that has been widely investigated for medium-grade heat storage application is magnesium hydroxide/oxide (Mg(OH)_2_/MgO) [2]. However, according to Kato et al. [3,4] this material has a relatively high dehydration temperature, which limits its full potential. Furthermore, it has a low thermal conductivity [5], which can affect the thermal efficiency of TCES systems.

To address the issue of high dehydration temperature, various methods [6,7] have been proposed and investigated. For instance, Shkatulov et al. [8] reduced the dehydration temperature of Mg(OH)_2_ by 50 °C by doping it with sodium nitrate (NaNO_3_). Furthermore, Shkatulov and Aristov [9] doped Mg(OH)_2_ with lithium nitrate (LiNO_3_) and obtained a reduction of 76 °C in the dehydration temperature. Sun et al. [10] decreased the dehydration temperature of Mg(OH)_2_ with cerium nitrate (Ce(NO_3_)_3_) by 29 °C. Li et al. [11] reduced the dehydration temperature of Mg(OH)_2_ by 56 °C with 10 wt% LiNO_3_ as a dopant. In our previous work [12], we achieved a reduction of 23 °C in the dehydration temperature and an increase of 6% in the heat storage capacity of Mg(OH)_2_ with 5 wt% potassium nitrate (KNO_3_).

However, the issue of low thermal conductivity remains largely unresolved, though the incorporation of nanomaterials has been proposed as a potential solution to enhance it [13]. These nanomaterials must possess certain properties, such as high thermal conductivity, compatibility with the host material, thermal stability, and a large surface area to be effective [14]. In this regard, Chen et al. [15] and Gollsch et al. [16] used silicon and aluminum oxides (SiO_2_ and Al_2_O_3_) in TCES materials. However, SiO_2_ was observed to have negatively impacted the heat capacity of the storage material [15]. Coetzee et al. [17] reviewed the effect of nanomaterials on the thermal conductivity of composite materials and concluded that nanoparticles, such as alumina (Al_2_O_3_), could create thermally conductive pathways within the matrix, allowing for efficient heat conduction. These nanoparticles exhibit stability across a wide range of temperatures without degrading. However, careful consideration of the dosage was advised to prevent potential adverse effects.

For this reason, Al_2_O_3_ was proposed for thermal conductivity enhancement of the developed KNO_3_-doped Mg(OH)_2_ energy storage material [12]. It is proposed to utilize a molecular dynamics (MD) simulation approach to analyze and interpret the thermophysical and structural behavior of the composite material at atomic and molecular levels. Specifically, MD simulations using the LAMMPS (Large-Scale Atomic/Molecular Massively Parallel Simulator) code have the capability of determining the trajectories of particle interactions within composite materials and a reliable means of acquiring targeted thermodynamic information. For instance, Xu et al. [18] simulated the agglomeration behavior of a CaO/Ca(OH)_2_ system and found that agglomeration occurred more slowly during discharging cycles and that adding silica (SiO_2_) particles to CaO further reduced agglomeration. In another study, Shkatulov et al. [19] used MD simulations to investigate metastability in a nitrate-doped Mg(OH)_2_ system and the role of molten nitrate interfaces in enhancing reversibility.

Therefore, MD simulations and experimental approaches were employed to evaluate the thermal performance of the developed Mg(OH)_2_-KNO_3_ material.

## 2. Results and Discussion

### 2.1. MD Simulation Results

#### 2.1.1. Dose Determination

**Figure 1:**
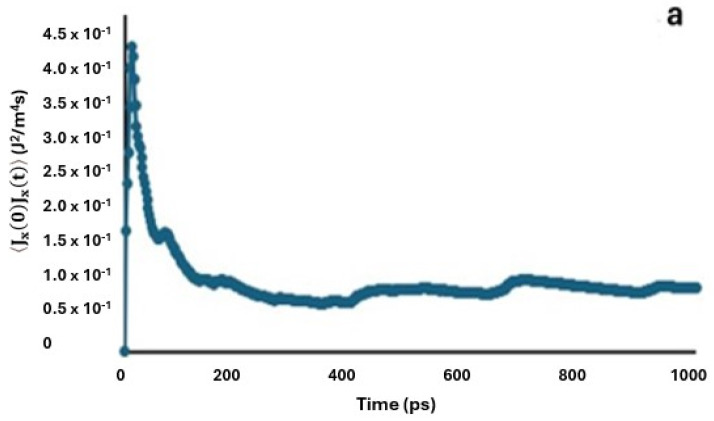

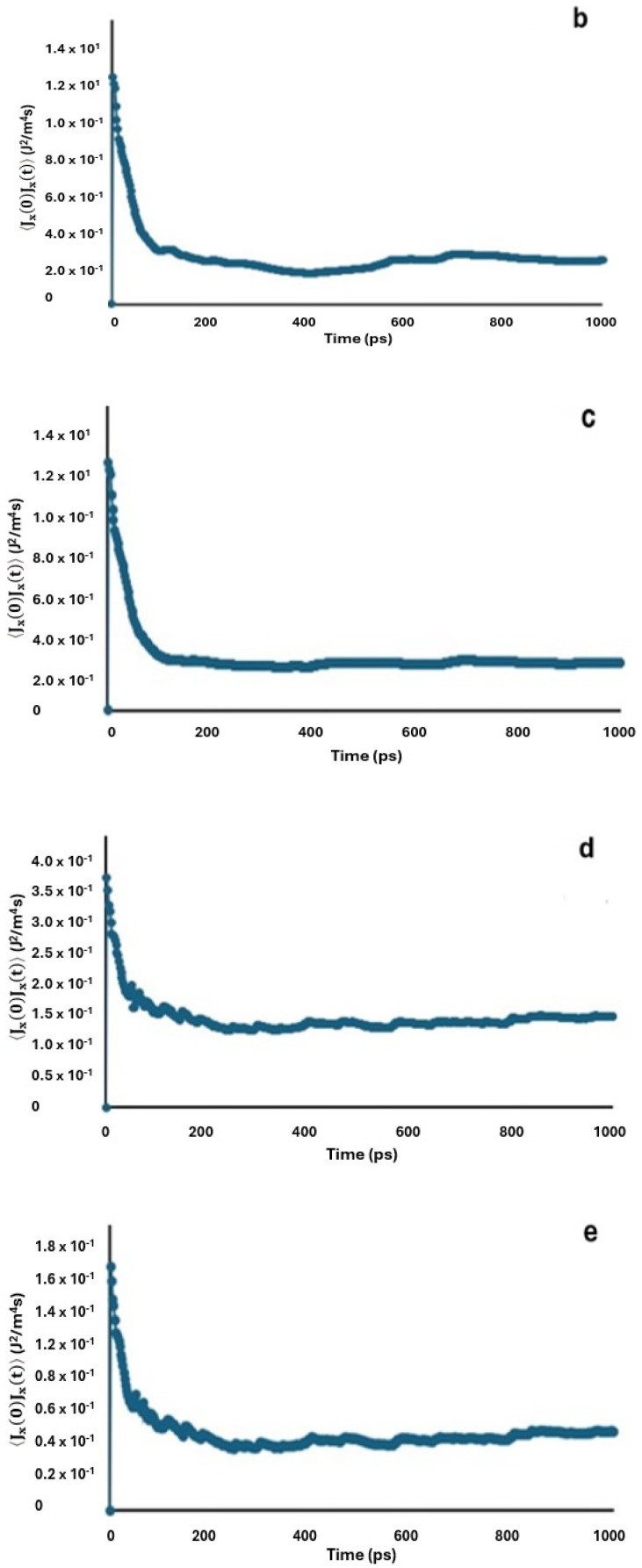
Profiles of the autocorrelation function over time for (**a**) MH-PN5, (**b**) MH-PN5AO5, (**c**) MH-PN5AO10, (**d**) MH-PN5AO15, and (**e**) MH-PN5AO20.

In Equation (4), the autocorrelation function of the heat flux, $\langle J_{x}(t)J_{x}(0)\rangle$, was shown to describe how the heat flux at time t is related to the heat flux at time t = 0. Figure 1 shows the graphical profiles of the autocorrelation function over time.

It can be seen from Figure 1 that the autocorrelation function generally starts at a maximum value when t = 0 and decays over time t. Thus, the function approximates an exponential decay model, ⟨J_x_(t)J_x_(0)⟩∼e^−t/τ^, where τ is the correlation time. The thermal conductivity is calculated as the integral of the function over time. That is, the area under the curve corresponds to the value of κ. The initial value and the shape of the curve determine how quickly the thermal conductivity integral converges. In Figure 1a, the function decays from a value of 0.437 to 0.0709 J^2^/m^4^s with a calculated decay constant of 1.82 ps^−1^. The decay constant can be defined as the inverse of the correlation time τ and characterizes the rate of an exponential decay process. Thus, it represents how quickly a quantity decreases over time. Similarly, the functions in Figure 1b–e decayed from 1.22 to 0.171, 1.26 to 0.221, 0.372 to 0.127, and 0.165 to 0.042 J^2^/m^4^s, respectively, with decay constants 1.96 ps^−1^, 1.74 ps^−1^, 1.07 ps^−1^, and 1.37 ps^−1^. The higher the decay constant, the faster the decay rate of the autocorrelation function. Conversely, if the decay constant is small, the decay rate of the autocorrelation function is slow. This has implications for the interpretation of the results as follows.

It was observed, however, that the autocorrelation functions in Figure 1 did not converge to zero at large t. This is typically due to insufficient equilibration or background noise in a simulation with a finite time frame. Since the system was adequately equilibrated, the observed behaviour was likely attributed to background noise. To address this, each background value was calculated by averaging the autocorrelation function over a time range where the function no longer exhibited a clear decay, and this value was then subtracted from the calculated thermal conductivity. 

**Figure 2:**
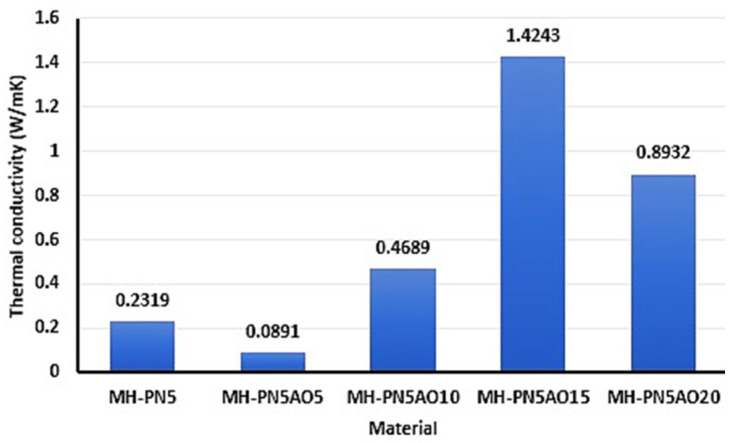
Thermal conductivity of the doped samples and the samples with nanomaterial added.

The results of the thermal conductivities of MH-PN5 and the various proportions of nano-Al_2_O_3_-added materials obtained by EMD simulations are presented in Figure 2.

It can be seen in Figure 2 that by increasing the MH-PN5/Al_2_O_3_ ratios, the thermal conductivities varied considerably in comparison with the MH-PN5 sample. This agrees with the proposition of Coetzee et al. [17] that the addition of nanoparticles to a material could increase or decrease its thermal conductivity depending on the dosage. The thermal conductivity of the doped material with 15 wt% Al_2_O_3_ addition (that is, MH-PN5AO15) showed the highest thermal conductivity value of 1.4243 W/mK compared with 0.0891, 0.4689, 0.8932, and 0.2319 W/mK for MH-PN5AO5, MH-PN5AO10, MH-PN5AO20, and MH-PN5, respectively. In the context of the G-K theory, the autocorrelation function $\langle J_{x}(t)J_{x}(0)\rangle$ decays more slowly in the MH-PN5AO15 sample, as previously seen in the lowest decay constant value of 1.07 ps^−1^, meaning that heat flux fluctuations persist longer. This indicates that the material has more efficient energy transfer mechanisms, allowing heat flux to remain correlated over longer times, thus facilitating better heat conduction.

#### 2.1.2. Enhanced Doped Material

**Figure 3:**
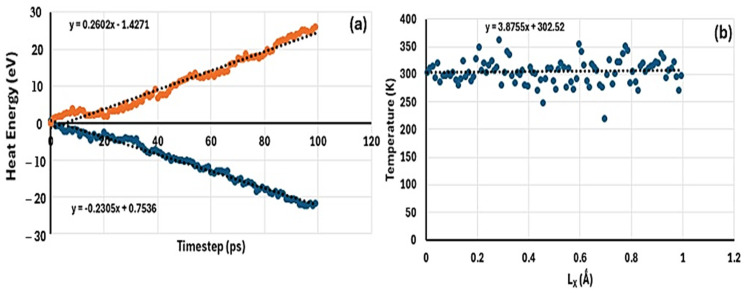
A plot of (**a**) heat energy against timestep to derive heat flux, (**b**) temperature against coordinate to derive temperature gradient for MH material.

The NEMD results for comparison of thermal conductivity of samples MH, MH-PN5, and MH-PN5AO15 are determined as follows. For the pure MH, the plot to obtain the heat flux is shown in Figure 3a. The heat flux value was calculated as the average of the absolute values of the slopes given in the two fitted line equations (inset), representing the heat extracted from the hot (pink) and the heat added to the cold (blue) regions. This gives a value of 0.2454. On the other hand, Figure 3b represents the temperature gradient as a plot of the temperature averaged over all the chunks of the simulation box, across the direction of heat transmission. Here, the value is 3.8755, the absolute value of the slope of the fitted line represented by the line equation (inset). Using Equation (6), the thermal conductivity was obtained as 0.0633 W/mK. To evaluate the variability around the target temperature (293 K), a standard error of 2.2374 was obtained for Figure 3b. The small standard error value indicates that the data points were fairly representative of the true mean temperature. It can be seen that more data points cluster closer to the line than away.

**Figure 4:**
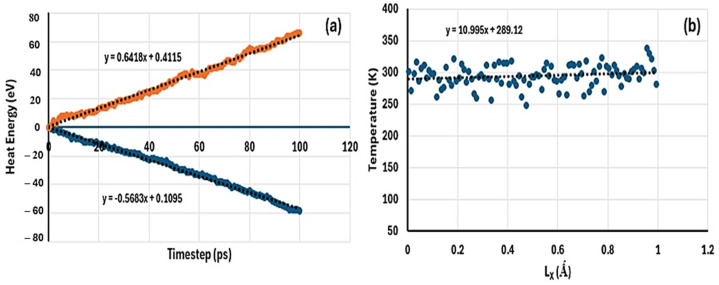
A plot of (**a**) heat energy against timestep to derive heat flux, (**b**) temperature against coordinate to derive the temperature gradient for MH-PN5 material.

Similarly, the plots for deriving the heat flux and temperature gradient for the doped material (MH-PN5) are shown in Figure 4. For the heat flux value, the average of the absolute values of the slopes of the fitted lines in Figure 4a gives 0.6051, whereas the coefficient for the temperature gradient curve in Figure 4b gives 10.9950. Therefore, using Equation (6) results in a thermal conductivity of 0.0550 W/mK. A standard error value of 1.7373 was obtained for the scatter graph (Figure 4b), indicating a small variability in the data points. It is observed that the thermal conductivity of the doped material (MH-PN5) is slightly lower than that of the pure magnesium hydroxide material (MH) by 0.0083 W/mK.

**Figure 5:**
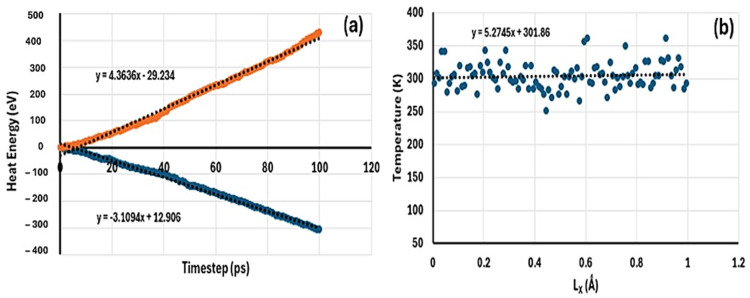
A plot of (**a**) heat energy against timestep to derive heat flux, (**b**) temperature against coordinate to derive the temperature gradient for MH-PN5AO15 material.

For the enhanced material (MH-PN5AO15), the heat flux and temperature gradient were deduced from Figure 5a and Figure 5b respectively, wherein the thermal conductivity is calculated as 0.7084 W/mK. Here, a standard error value of 2.0583 was obtained for Figure 5b, showing a small variability in the data distribution around the line. Therefore, by comparison, the theoretical thermal conductivity of MH-PN5AO15 is 0.6534 W/mK higher than that of the MH-PN5 material. This shows that the addition of Al_2_O_3_ nanomaterials has the potential to enhance the thermal conductivity of the doped material.

### 2.2. Experimental Results

#### 2.2.1. XRD

**Figure 6:**
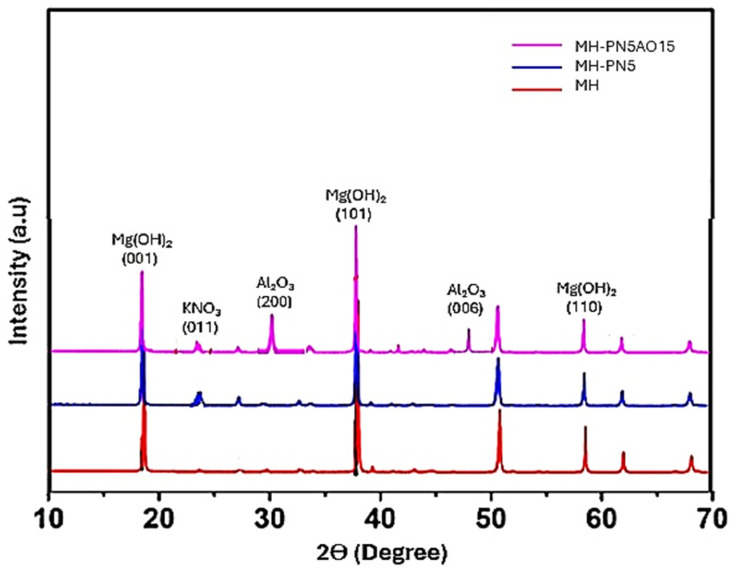
XRD spectra of MH, MH-PN5, and MH-PN5AO15.

The synthesized materials were analyzed for phase identification of the composite elements using X-ray diffraction (XRD). The XRD spectra for pure Mg(OH)_2_ (MH), doped Mg(OH)_2_ (MH-PN5), and enhanced Mg(OH)_2_ (MH-PN5AO15) are shown in Figure 6. MH exhibited a single phase (peak), whereas the composites MH-PN5 and MH-PN5AO15 showed two distinct peaks corresponding to Mg(OH)_2_ and KNO_3_, with MH-PN5AO15 additionally displaying a third peak for Al_2_O_3_. The observed planes match the standard Powder Diffraction File (PDF) from the Inorganic Crystal Structure Database (ICSD), with references 01-074-2220 for pure Mg(OH)_2_ brucite, 00-001-0493 for standard KNO_3_, and JCPDS card No. 35-0121 for Al_2_O_3_.

These findings demonstrate the successful incorporation of KNO_3_ and Al_2_O_3_ into the Mg(OH)_2_ matrix, resulting in composite materials with distinct phases. The presence of multiple peaks in the XRD spectra indicates that the doping process did not alter the fundamental structure of Mg(OH)_2_ but revealed additional phases corresponding to the dopants. By carefully analyzing the XRD spectra and matching the observed peaks to standard references, the study confirms the successful synthesis via phase identification of the composite materials. This step is crucial for validating the effectiveness of the doping process and ensuring the desired material properties for further analysis.

#### 2.2.2. Thermal Conductivity

The results of thermal conductivity measurements for the three materials are summarized in Table 1.

**Figure 7:**
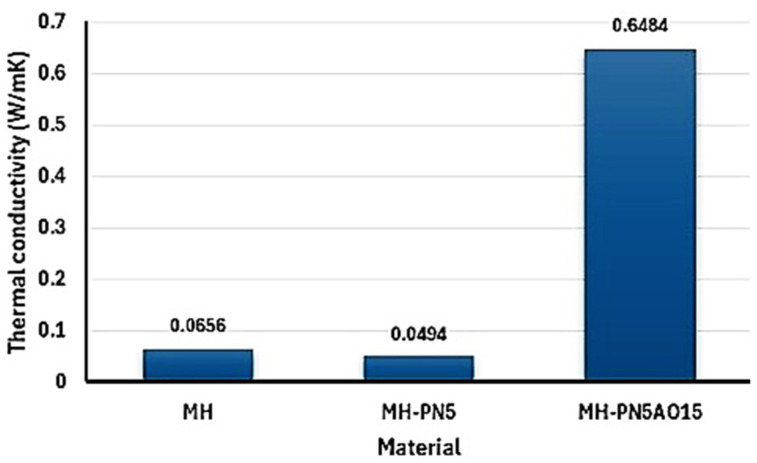
Chart showing the experimental thermal conductivity values of the materials.

As shown in Table 1, the small values of the standard error signify that the sample mean was a reliable estimate of the measurements, and the narrower confidence interval indicates a higher confidence in the measured parameters. Therefore, it is reasonable that the thermal conductivity values lie within a good accuracy range. The thermal conductivities of the materials are presented in Figure 7. As seen in Figure 7, the thermal conductivity of the pure magnesium hydroxide material (MH) is 0.06562 W/mK. Doping MH with KNO_3_ (as in MH-PN5 material) appears to have a small impact on the thermal conductivity of the MH as the thermal conductivity of the doped material is 0.0494 W/mK. However, incorporating nano-Al_2_O_3_ (MH-PN5AO15) enhanced the thermal conductivity of the doped material by 0.5990 W/mK. This is likely due to the well-dispersed Al_2_O_3_ nanoparticles forming an efficient network within the composite matrix. This configuration could have created sufficient thermal conduction pathways, similar to what has been seen in other polymer composite studies [20]. Furthermore, the alumina nanoparticles may have facilitated phonon transport by providing high-conductivity routes, reducing phonon scattering at grain boundaries.

Table 2 shows a comparison of the theoretical and experimental results of the thermal conductivity of the materials.

As seen in Table 2, the theoretical value for MH is lower than the experimental value, with an error margin of 4%. The marginal discrepancy suggests that the theoretical model for this material is fairly accurate. However, for MH-PN5 and MH-PN5AO15 materials, the theoretical values are higher than the experimental values, with 10% and 9% errors, respectively. This indicates that the theoretical models for these materials are fairly accurate but still have room for improvement. The discrepancies could arise from several factors such as simplifications or assumptions in the theoretical model that do not fully capture the complexities of the material’s microstructure (complex interactions between the multiple components of the materials). For instance, theoretical models often require parameters (such as force field potentials or lattice constants) that may be estimated or taken from the literature, which could potentially introduce inaccuracies. Additionally, experimental errors could contribute to this difference. Overall, the theoretical results show reasonable agreement with the experimental measurements, suggesting that the theoretical approach is generally valid but may require refinement to account for more complex interactions.

## 3. Methods and Materials

### 3.1. MD Simulation Approach

MD simulation is a computational technique used to study the physical movements of atoms or molecules in a system over time. By solving Newton’s equations of motion for a system of interacting particles, MD simulations provide insights into materials’ dynamic behavior and properties at the atomic level [21]. This approach allows researchers to predict and analyze materials’ thermal, mechanical, and transport properties, which are often challenging to measure experimentally [22].

In this work, an average of 11,000 atoms were randomly distributed in a 50 × 50 × 50 Ǻ^3^ periodic cubic cell using the PACKMOL package [23]. The periodic boundary condition minimizes edge effects in the finite-sized simulation box and approximates an infinite system in real-world situations. It is built on the concept that when a particle crosses one boundary of the simulation box, it re-enters the box from the opposite side, creating the illusion of a continuous, infinite system.

Classical MD simulations were performed using the LAMMPS code (Sandia National Laboratories, Albuquerque, NM, USA) version 2020 to perform a series of MD simulations of the doped and enhanced materials. Initially, the KNO_3_-doped Mg(OH)_2_, designated as MH-PN5, was simulated with varying proportions (5, 10, 15, and 20 wt%) of Al_2_O_3_, designated as MH-PN5AO5, MH-PN5AO10, MH-PN5AO15, and MH-PN5AO20, respectively, to find the optimal dose of the nanomaterials in terms of the thermal conductivity of MH-PN5.

**Figure 8:**
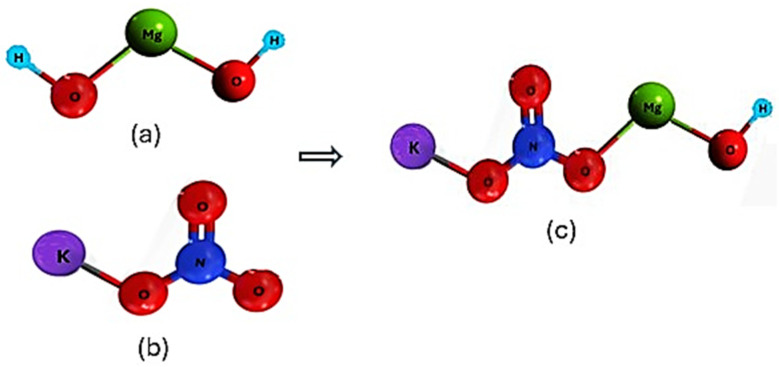
The molecular structures of (**a**) Mg(OH)_2_ (**b**) KNO_3_, and (**c**) KNO_3_-doped Mg(OH)_2_.

**Figure 9:**
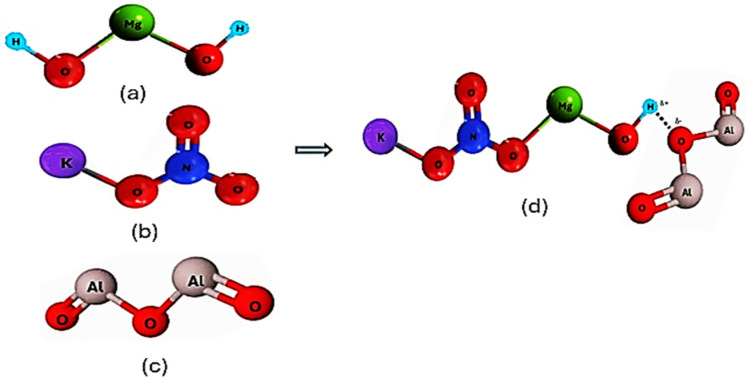
The molecular structures of (**a**) Mg(OH)_2_ (**b**) KNO_3_, (**c**) nano-Al_2_O_3_, and (**d**) the enhanced KNO_3_-doped Mg(OH)_2_.

**Figure 10:**
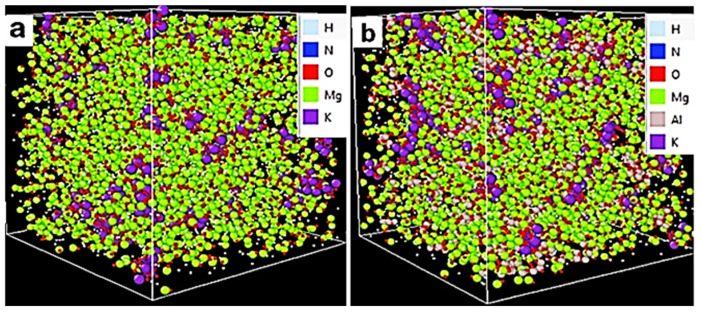
Initial configuration of (**a**) MH-PN5 and (**b**) MH-PN5AO15.

Figure 8 shows the schematic view of the interaction between constitutive atoms of Mg(OH)_2_, KNO_3_, and the KNO_3_-doped Mg(OH)_2_. Figure 9 represents the schematic of the interaction between constitutive atoms of Mg(OH)_2_, KNO_3_, nano-Al_2_O_3_, and the enhanced doped material. These interactions between the components are likely to be more complex and could involve various chemical and physical forces. However, Mg(OH)_2_ and Al_2_O_3_ (Figure 9) may have more significant direct interactions because the hydroxide can interact strongly with alumina due to its hydroxyl groups. In any case, we only tried to make sense of the possible complicated interactions in the given situation. The initial configurations of the randomly distributed atoms in the simulation box with periodic boundary conditions are shown in Figure 10. Specifically, the initial configuration of MH-PN5 (Figure 10a) illustrates the distribution of atoms within the 5 wt% KNO_3_-doped Mg(OH)_2_ matrix. The green, red, and purple spheres represent Mg, O, and K atoms, respectively, while the light-blue and dark-blue spheres represent H and N atoms, respectively. In MH-PN5AO15 (Figure 10b), the addition of nano-aluminum oxide (Al_2_O_3_) is depicted. Here, the introduction of Al atoms is indicated by the grey spheres, which are dispersed throughout the matrix. This configuration highlights the distribution and potential interaction sites of the nano-aluminum oxide within the Mg(OH)_2_/KNO_3_ composite, aiming to enhance its thermal conductivity.

#### 3.1.1. Interatomic Potential

The Lennard-Jones (L-J) potential V(r) expressed in Equation (1) was employed for the calculation of pairwise interactions between the atoms in the doped and enhanced materials. The force field (potential) was crucial in determining how particles moved over time, thus influencing the calculation of dynamic properties. It also defined the system’s potential energy, guiding it to a stable equilibrium configuration during the simulation’s energy minimization and equilibration phases.

(1) $$V\left(r\right)=\frac{Cq_{i}q_{j}}{er_{ij}}+4\varepsilon \left[{\left(\frac{\sigma }{r_{ij}}\right)}^{12}-{\left(\frac{\sigma }{r_{ij}}\right)}^{6}\right]$$

where C is the energy conversion constant, q_i_, and q_j_ are the charges on atoms of types i and j, respectively, e is the dielectric constant, r is the distance between atoms, ε is the potential well depth, and σ is the finite distance at which the interatomic potential is zero. Equation (1) is made up of two parts: the Coulombic potential (first term) and the Lennard-Jones potential (second term).

The parameters for the electrostatic and interatomic interactions used in this study are taken from the literature [24,25,26] for similar systems and are provided in Table 3.

In this simulation, a dielectric constant of 4.0 was employed, which effectively reduced the Coulombic interactions to 25% of their default value (1.0). This value was selected after testing different dielectric constants, wherein higher or lower values led to computational instability, specifically the ‘lost atoms’ error. Thus, a dielectric constant of 4.0 was found to be most suitable for maintaining both the accuracy and stability of the model.

The Lennard-Jones potential is ideal for such large systems due to its simplicity and low computational cost. Additionally, it uses the Lorentz–Berthelot rules (Equations (2) and (3)) to estimate the cross-term interactions between unlike species.

(2) $$\sigma_{ij}=\frac{1}{2}\left(\sigma_{i}+\sigma_{j}\right)$$

(3) $$\varepsilon_{ij}=\sqrt{\varepsilon_{i}\varepsilon_{j}}$$

#### 3.1.2. Simulation Details

Once the forces acting on particles were known, classical Newton’s law of motion was automated to obtain particle trajectories via a finite difference scheme by an integration method known as Verlet integration. The MD was performed in the isothermal-isobaric (NPT) ensemble using a Nosé–Hoover thermostat with a timestep of 1 fs. A timestep between 1 and 2 fs (shorter than the fastest movements in the molecules) is recommended for this type of atomistic simulation [27]. As recommended in the LAMMPS documentation [28] as a good choice for such models, the temperature and pressure coupling constants used were 0.1 ps and 1 ps, respectively. To achieve a more realistic and stable configuration, the initial step involved optimizing the structures through a series of temperature changes. Initially, the system was heated from 293 K to 823 K over 2 × 10^6^ steps using an NPT ensemble, followed by cooling back to 293 K under the same conditions. Subsequently, an energy minimization process was conducted to redistribute the atoms, aiming for a configuration with minimal energy.

**Figure 11:**
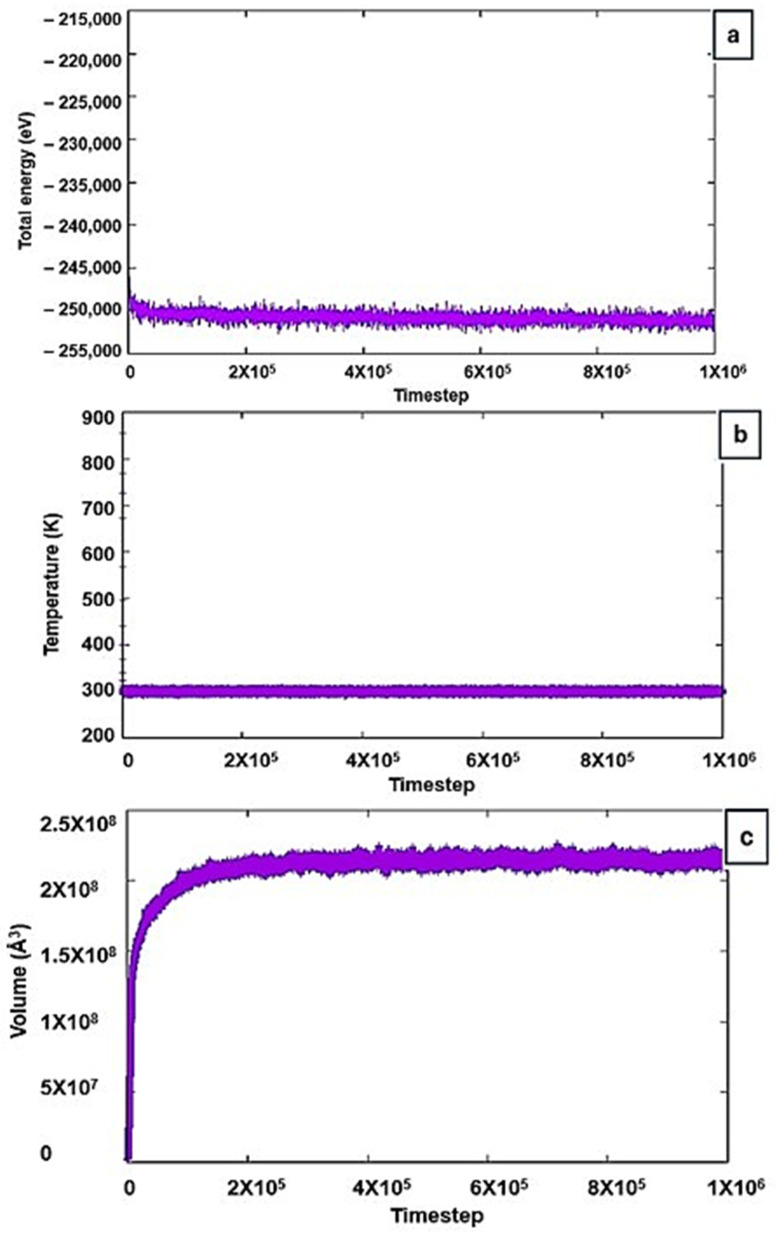
Stable equilibrium profiles for (**a**) total energy, (**b**) temperature, and (**c**) volume.

These iterative procedures were crucial for resetting the system’s initial configuration and resolving any potential atom overlaps, ensuring more accurate simulations. Following this, the system underwent an equilibration run at 293 K, until reaching a relatively stable density. At equilibrium, the system’s thermodynamic potential reaches a minimum, indicating a balanced state of energy and interactions within the system. To check for this, the energy was plotted against time (or run steps) to observe when the energy becomes largely stable, as shown in Figure 11a. The energy stabilization around −250,000 eV signifies that the system’s atoms have settled into a low-energy configuration, where their interactions are balanced. This value indicates the cumulative potential energy of all interactions within the system (including van der Waals forces and electrostatic interactions). Furthermore, the temperature and volume profiles in Figure 11b,c also show stability over 1,000,000 timesteps, confirming that the system was adequately equilibrated before proceeding with further simulations. The use of specific plotting software allowed for detailed analysis of the energy vs. time data, confirming the system’s readiness for further simulation steps.

**Figure 12:**
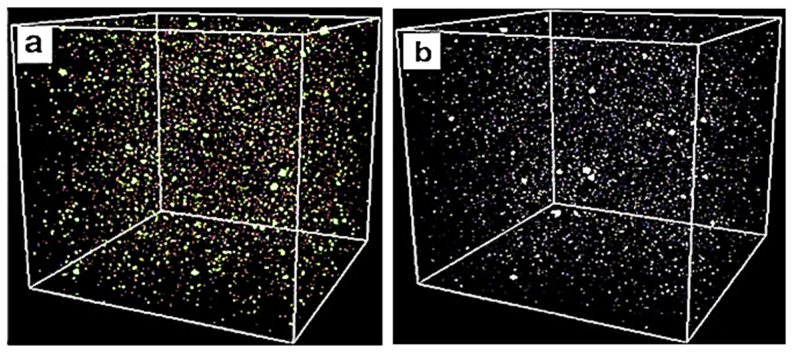
The optimized configuration of (**a**) MH-PN5 and (**b**) MH-PNAO15.

Figure 12 displays the optimized configurations of the simulated materials. In Figure 12a, the configuration of MH-PN5 (5 wt% KNO_3_-doped Mg(OH)_2_) is shown. The atoms are distributed uniformly throughout the cubic cell, reflecting a stable and homogeneous mixture. Similarly, Figure 12b displays the optimized configuration of MH-PN5AO (Mg(OH)_2_/KNO_3_ with the addition of nano-Al_2_O_3_) also showing atoms well dispersed within the matrix, indicating good mixing and interaction with the base material. The densities of MH-PN5 and MH-PN5AO15 optimized structures were 2.29 and 2.64 g/cm^3^, respectively, each obtained as an average over the entire simulation time. The higher density of MH-PN5AO15 material obtained by doping Al_2_O_3_ into the composite may be attributed to the Al_2_O_3_ particles filling voids within the structure and improving the overall packing efficiency.

**Figure 13:**
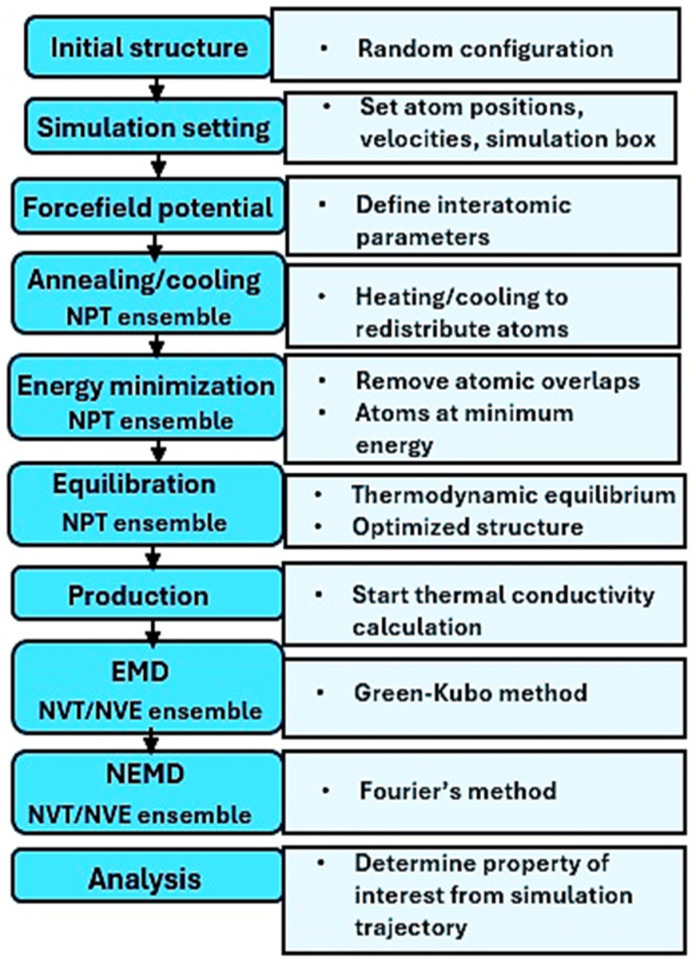
Flowchart of the MD simulation procedure used in this work.

The MD simulation protocol followed in this work is summarized in Figure 13, in which the process began with preparing the initial structure and defining the simulation settings and force field parameters. The structures were annealed, minimized, and equilibrated to optimize them before the production runs to gather data for analysis.

##### Thermal Conductivity Calculation

Stage 1: EMD Method

The optimal dose of nanomaterials added to the doped material was calculated using Equilibrium Molecular Dynamics (EMD), employing the Green–Kubo (G-K) method. The G-K method in Equation (4) calculates the thermal conductivity κ directly from the ensemble-averaged autocorrelation of the x component of the microscopic heat current (J_x_) that occurs during the simulation.

(4) $$\kappa =\frac{1}{K_{B}T^{2}V}\int_{0}^{\infty }\langle J_{x}(t)J_{x}(0)\rangle dt$$

where V is the system volume, T is temperature, K_B_ is the Boltzmann constant, and 〈J_x_(0) J_x_(t)〉 indicates the ensemble average. Thermal conductivity in the G-K method is related to the elapsed time, which dissipates the fluctuations [29]. The EMD simulation was performed following the method described by Alexander et al. [30]. A small NVT equilibration run was performed for 100 ps with the help of a Nosé–Hoover thermostat to relax the system before calculating the thermal conductivity. An NVE simulation was then performed to thermalize the system by allowing the evolution of phonons. By removing the thermostat and barostat, atomic motions were freed from artificial rescaling, enabling a more realistic equilibration before thermal conductivity computation.

As mentioned earlier, the G-K method is based on linear response theory and relates thermal conductivity directly to the microscopic heat current fluctuations. Additionally, unlike NEMD methods, the G-K approach does not require reaching a steady-state heat flow, which often demands longer simulations. Due to its straightforward process for calculating thermal conductivity, the G-K method was employed as an initial screening tool to efficiently narrow down the number of composite materials for further simulation.

Stage 2: NEMD Method

The Non-Equilibrium Molecular Dynamics (NEMD) method was then employed to estimate the thermal conductivity of the material sample with the optimal nanomaterial dose obtained from stage 1 above. The NEMD method was employed at this stage due to its analogy to the transient hot wire (THW) method used in this study’s experimental thermal conductivity measurements. Specifically, the analogy is that both techniques rely on creating a temperature gradient and subsequent heat flow analysis to determine thermal conductivity. Furthermore, the fundamental principle in both methods is Fourier’s law used to relate the thermal conductivity to the observed heat transfer characteristics.

The NEMD method is a steady-state approach in which thermal conductivity κ is calculated from the steady-state heat flux J through the material and the resulting temperature gradient ∇T, expressed as

(5) $$\kappa =-\frac{J}{\nabla T}$$

At first, the system was simulated using the NVT ensemble in 10,000 steps for thermalization. Next, the simulation cell was divided into three distinct regions to establish a thermal gradient: a central region and two boundary regions. The boundary regions were maintained at different temperatures using Langevin thermostats, creating a heat flux from the hot region to the cold region. The NVE (constant number of particles, volume, and energy) ensemble was utilized for 50 ps in the central region whilst heat was added to the hot region and removed from the cold region, facilitating a continuous heat flow through the system.

Once the system reached a non-equilibrium steady state, the heat flux J and the resulting temperature gradient ∇T were derived and the thermal conductivity κ was determined based on Fourier’s law of heat conduction.

The heat flux was calculated from the rate of heat exchange between the hot and cold regions. This was done by extracting the data from the output file for both regions and plotting them on the same axes, as demonstrated in the work of Winczewski and Muna [31]. The average of the two slope values (absolute) of the curves gave the heat flux. A linear fit was performed on the temperature profile along the direction of heat flow within the central region to obtain the temperature gradient. This direct calculation method allowed for an assessment of the thermal conductivity by simulating the microscopic interactions and transport processes inherent to the material system.

### 3.2. Experimental Procedure

#### 3.2.1. Material Development

**Figure 14:**
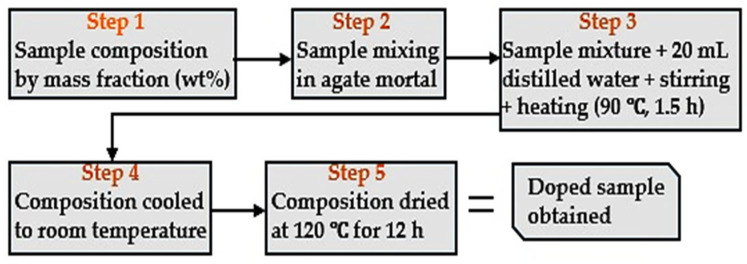
Flow diagram for the doping procedure used.

The materials used were 95.0% purity magnesium hydroxide, Mg(OH)_2_, 99.0% purity potassium nitrate, KNO_3_, and nano-aluminum oxide (alumina), Al_2_O_3_ (all purchased from Sigma-Aldrich, Gillingham, UK). The KNO_3_-doped Mg(OH)_2_ was prepared by incorporating 5 g KNO_3_ into 95 g Mg(OH)_2_, representing the optimal 5 wt% KNO_3_. Initially, the KNO_3_ and Mg(OH)_2_ powders were carefully blended using an agate mortar to ensure a homogeneous mixture. The mixture was then transferred to a beaker for liquid blending with 20 mL of distilled water. The blend was continuously stirred at a moderate speed and heated at 90 °C for 1.5 h. Subsequently, the composition was allowed to cool to room temperature before undergoing drying in an oven at 120 °C for 12 h to obtain the synthesized composite. Figure 14 represents the summary of the steps in the doping procedure.

**Figure 15:**
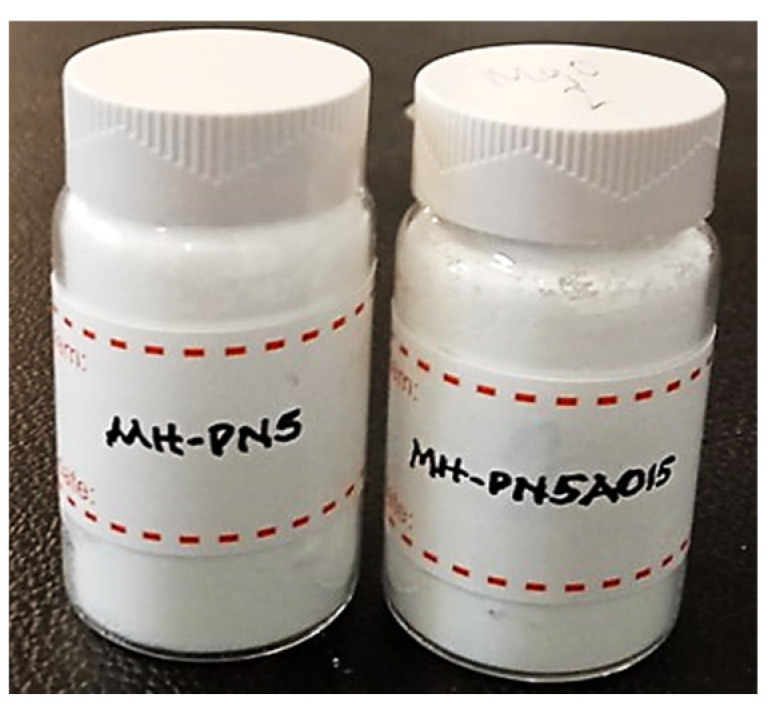
A photo showing the doped and enhanced materials.

For the enhanced material, the optimal proportion of Al_2_O_3_, estimated in the EMD simulation, was added to the Mg(OH)_2_/KNO_3_ material following the same procedure as previously described. For ease of reference, the labels MH-PN5 and MH-PN5AO represent the KNO_3_-doped and Al_2_O_3_-enhanced samples. respectively. Samples of the developed materials are shown in Figure 15.

#### 3.2.2. Powder X-Ray Diffraction

Powder X-ray diffraction (XRD) analysis was conducted to identify the composition of phases in the developed composite materials. This investigation was carried out at room temperature using a PANalytical X’Pert PRO diffractometer, which utilized CuKa radiation (with a wavelength of λ = 1.5406 Å, operating at 40 kV and 40 mA). The scanning process involved incrementing the 2-Theta values by a 0.02 step size, each lasting 50 s, spanning the range from 2° to 70°.

#### 3.2.3. Thermal Conductivity Measurement

**Figure 16:**
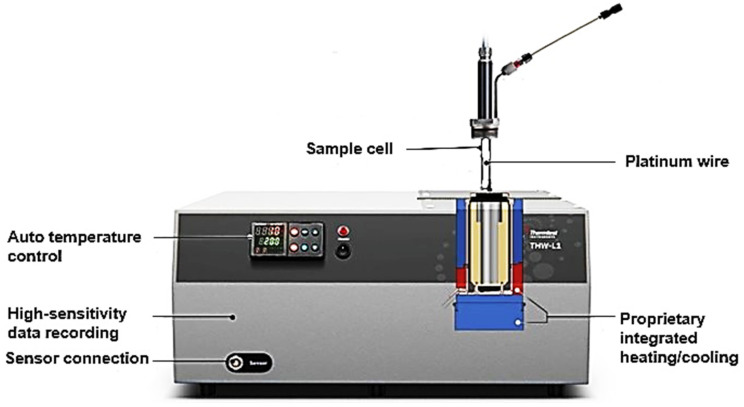
Thermtest THW-L1 thermal conductivity meter used for the measurements.

The thermal conductivities of the samples were measured using the Thermtest transient hot wire (THW-L1) thermal conductivity meter (shown in Figure 16). Initially, the samples were dried in the oven at 110 °C for 6 h to drive off any moisture that could affect measurements and allowed to cool to room temperature. Each sample was then filled into the instrument’s sample holder and the sensor wire (a thin platinum heating wire) was completely inserted into the sample. The sensor wire was heated (using a constant current source) to 20 °C as specified in the materials’ datasheets for characterization. The instrument’s software then calculated and displayed the thermal transfer through the samples. To ensure the reliability of the results, each sample was tested 10 times, and a standard error was determined at a 95% confidence level. Measurements were taken at 20 °C (room temperature) and with 15 min between each of the 10 experiments.

Subsequently, the percentage error between the theoretical (MD simulated) and the experimental values was determined using the formula

(6) $$Percentage\;Error=\left|\frac{Theoretical-Experimental}{Theoretical}\right|\times 100$$

## 4. Conclusions

This study investigated the enhancement of thermal conductivity in KNO_3_-doped Mg(OH)_2_ for medium heat storage, utilizing molecular dynamics (MD) simulations. The MD simulations revealed that the incorporation of nanomaterials like Al_2_O_3_ could lead to variable outcomes depending on the dose. In this case, an optimal dose of 15 wt% Al_2_O_3_ significantly improved the thermal conductivity of the composite (doped) material. Further evaluation showed that the thermal conductivity of the doped material (MH-PN5) was 0.0550 W/mK, which is a decrease of 0.0083 W/mK compared with 0.0633 W/mK of the pure material (MH). However, the incorporation of nanomaterials (MH-PN5AO15 variant) significantly increased the value to 0.7084 W/mK, which is an increase of 0.6534 W/mK over MH-PN5, showcasing the potential of this approach for enhancing heat transfer in these materials.

Experimental results showed good agreement with the theoretical results within an error margin of ≤10%, thus suggesting that this systematic evaluation validates the accuracy of MD simulations in predicting this thermal property. Future work will focus on kinetic studies of the material to better understand its performance for efficient energy storage. Additionally, combining atomic positions from MD simulations with the first Born approximation can help generate XRD spectra that reflect the evolution of atomic arrangements under varying conditions.

## Figures and Tables

**Table 1:** Results of experimental measurement of thermal conductivity for the materials.

Material	MH	MH-PN5	MH-PN5AO15
Mean	0.06562	0.04936	0.64841
Standard deviation	0.0008661	0.0000549	0.0099096
Standard error	0.0002739	0.0000174	0.0031337
Confidence interval	±0.000537	±0.000034	±0.006142
Accuracy	0.06562 ± 0.000537	0.04936 ± 0.000034	0.64841 ± 0.006142

**Table 2:** Comparison of theoretical and experimental thermal conductivity results.

Material	Theoretical (W/mK)	Experimental (W/mK)	Percentage Error (%)
MH	0.0633	0.0656	4
MH-PN5	0.0550	0.0494	10
MH-PN5OA15	0.7084	0.6484	9

**Table 3:** Lennard-Jones parameters of the simulated materials.

Material	Atomic Pair	ε (eV)	σ (Ǻ)	Charge
Mg(OH)_2_	Mg-Mg	2.253319968	1.501	+2
	O-O	0.005020786	3.369	−2
	H-H	0.000867282	1.780	+1
KNO_3_	K-K	0.00433641	3.18833	+1
	N-N	0.0073719	3.10669	+0.95
	O-O	0.006938258	3.00939	−0.65
Al_2_O_3_	Al-Al	0.00173457	4.053	+1.5
	O-O	0.009887018	2.860	−1.0

## Data Availability

All the data is available in this manuscript.
